# PFAS raise their ugly head in malaria control: Diverging views on risk substitution

**DOI:** 10.5281/zenodo.10807705

**Published:** 2024-03-12

**Authors:** Robert Bos

**Affiliations:** 1. Route du Creux-du-Loup 10, 1285 Athenaz, Canton Geneva, Switzerland.

## Abstract

A controversy has arisen over whether or not the replacement of PFAS compounds as a binder between insecticides and nets by other compounds has affected the nets' efficacy in preventing malaria transmission. Robert Bos places this matter in a broader and historical context and concludes that now is the time to revisit earlier concepts and provide sustainable malaria prevention and control with a broader foundation aiming for truly resilient results. The need to promote institutional arrangements conducive to inter-sectoral action is as great in WHO Member States as it is within the structure of the World Health Organization itself. Robert Bos is former Executive Secretary, WHO/FAO/UNEP/UN-Habitat Panel of Experts on Environmental Management for Vector Control, WHO, Geneva (1983-1995) former Scientist, Division of Environmental Health, later Department of Sustainable Development and Healthy Environments, later Department of Public Health and Environment, WHO, Geneva (1995-2009) and former Coordinator, Water, Sanitation and Health, WHO, Geneva (2009-2013).

The recent articles [1,2] about the controversy over the Vestergaard Long-Lasting Insecticide-Impregnated Nets (LLINs) and the possible impact a change in their coating formulation may have had on the increase in malaria incidence in Papua New Guinea (PNG) took me back to the late 1990s/early 2000s. At that time, I was part of the WHO delegation to the Intergovernmental Negotiating Committee (INC) meetings addressing the proposed Convention on the Reduction and Elimination of Persistent Organic Pollutants (POPs), later adopted and ratified as the Stockholm Convention on POPs [3]. Twelve POP chemicals were under discussion. Many of these were first-generation residual insecticides used during the WHO-led "global" malaria eradication campaign - global between inverted commas, because that campaign did not include most of Africa south of the Sahara, where colonial rule still dominated. At the time, the capacity in Africa to implement the campaign was considered insufficient. This was important for the way in which the POPs negotiations unfurled.

The hot issue on the agenda of the POPs INC was DDT - the environmental community (remember: the negotiations were carried out under the auspices of the United Nations Environment Pro-gramme) was adamant that DDT had to be phased out totally, including its use for public health purposes (e.g., malaria vector control). The arguments in favour of a total ban went back all the way to Rachel Carson's 1960s "Silent Spring” [4], and quoted evidence that the bio-accumulation of DDT in the food chain represented a growing toxicity risk in humans as well as updated information on the impact of DDT on biodiversity loss. It was also argued that restricting DDT use to indoor residual spraying for the control of anophelines could not be guaranteed and that with its continued public health use considerable amounts of DDT would flow into the agriculture sector, thereby contaminating the environment at large. In most countries DDT use for agricultural pest control had already been legally banned in the 1970s and 80s.

African countries, from their perspective, argued that they had never even had the benefit of using DDT for malaria vector control at the scale countries in Asia and Latin America had, and that they must not now be denied its use, in the face of the tremendous burden of malaria in their countries and particularly the high mortality among African children under five years of age. Other insecticides used for indoor residual spraying (IRS) were far more expensive and environmental management/ biological control were, by themselves, not a viable option for prevention and control in the eco-epidemiological settings of Africa.

The compromise agreed on to conclude the negotiations was, and I am paraphrasing, that countries could continue to use DDT for malaria vector control under the new Convention, provided that (1) they followed strict regulations proposed by the WHO for the safe handling, application and disposal of DDT; (2) they would, on an annual basis, report on DDT use for malaria control to the Secretariat of the POPs Convention and submit a request for the continued exoneration from the ban on its use; (3) they would take adequate measures to prevent the spill-over of DDT intended for malaria vector control into the agriculture sector; and (4) they would demonstrate, using agreed indicators, that a clear process of transition had been initiated and was making progress, from DDT use to a programme of integrated vector management (IVM) in which DDT would be gradually phased out. It would have to be ensured that such an IVM approach was at least as effective as the singular reliance on DDT.

This protocol has been in effect since the Stockholm Convention came into force in 2004 [5], and efforts to implement it have been supported mainly by the Global Environment Fund (GEF).

Fast forward to 2024. Globally, there is growing concern over the environmental and health impact of the group of Per- and Polyfluoroalkyl Substances (PFAS) - it contains literally thousands of compounds with a wide range of applications: from non-stick frying pans to the foams used by fire brigades to food-wrapping films to water resistant clothing to certain cosmetics. These manmade chemicals are all extremely persistent (they are referred to as "forever chemicals") and many bio-accumulate in the food chain. The level to which they end up in the environment depends on their use: there are measurements of high concentrations in surface water close to industrial sites where one or more PFAS compounds are produced and the areas surrounding airports are notorious contamination sites. There are reports from an increasing number of countries citing PFAS found in drinking water (for example: 45% of drinking water samples from across the USA contain PFAS, see: PFAS "forever chemicals" found in 45% of U.S. tap water, study says [6].

When it comes to health impacts due to exposure to PFAS, the picture is murky - studies suggest that certain PFAS compounds are carcinogenic, they may lead to immunosuppression and reduce the effectiveness of vaccines in children, and they may cause liver damage or high cholesterol levels. As is the case for many of these persistent compounds that may build up in fatty tissues over a lifetime of exposure, setting safety standards for concentrations in drinking water and food is challenging. Attribution is obscured by confounding factors over the long periods of exposure. And the uncertainty over public health impacts is demonstrated by the discrepancies between standards and norms set for acceptable PFAS concentrations in drinking water in different countries around the world.

And now we learn that one or more PFAS compounds are also used as binding factor in the production of insecticide impregnated mosquito nets. This comes just at a time of rising concern over the impacts of PFAS on environment and health. The relative hazard level of these LLIN-specific compounds will need to be assessed. The level to which this use leads to pollution of water resources requires further investigation. It is possible that there is point pollution at the LLIN production sites; it is also possible that regular washing of tens or hundreds of thousands of LLINs may be an important source of diffuse pollution. Any PFAS pollution that can be traced back to the production and use of LLINs must be put into context and compared to the various other sources of PFAS pollution. And the fact that the mortality of children under five attributable to malaria remains several orders of magnitude greater than that attributable to PFAS exposure must also be taken into consideration.

A controversy has arisen over whether or not the replacement of PFAS compounds as a binder between insecticides and nets by other compounds has affected the nets' efficacy in preventing malaria transmission. The company producing the LLINs, Vestergaard Frandsen, claims it responded to the concerns over PFAS risks by replacing it with another compound [2]. Bloomberg, a US-based, privately held financial, software, data and media company, published a report attributing the rise in malaria incidence in Papua New Guinea (PNG) to this change in formulation of the LLINs by Vestergaard, and suggesting that the real reason for the change may be economic - production using the new binding compound may lead to important savings [1]. Vestergaard, in its reaction [2], says that the nets with the new compound continue to meet the WHO standards of bio-efficacy and points to the various other factors that may have played a role in the resurgence of malaria in PNG: intrinsic factors of vector biology and ecology and external factors such as the effects of global climate change. It denies that the prime motivation for the change was economic.

The time seems ripe for independent, longitudinal, comparative studies of the bio-efficacy of LLINs produced with PFAS or with a substitute compound as the binder, over a full lifecycle (including regular washing) in a double-blind fashion and preferably in several eco-epidemiological settings. Such studies could be superimposed over already planned LLIN distribution activities. They may also include a component measuring how much PFAS ends up in the environment as a result of LLIN washing practices. The outcome of such studies should provide a solid evidence base for the decision making over the formulation of LLINs in terms of the insecticide binding compound used.

A few final words on the wider phenomenon of stagnation in the reduction of malaria incidence, or even its regression, as observed not only in PNG but in several other countries as well, beyond the question whether or not this can be attributed to the formulation and bio-efficacy of LLINs.

After the WHO Executive Board nominated Dr Brundtland in 1998 as the candidate of choice for the position of WHO Director-General, a transition team started working on a number of issues, including malaria prevention and control. A series of meetings was held to develop the blueprint of what was to become the Roll Back Malaria initiative. Two principles were established early on in this process: (1) the health sector would take back exclusive ownership of the malaria prevention and control efforts ("no more intersectoral collaboration") and (2) the malaria prevention and control strategy would be based on two pillars: case detection/drug treatment and the distribution of insecticide-impregnated mosquito nets (this went back to the outcome of the 1992 Amsterdam Conference on Malaria [7]). Asked for his views on intersectoral action for malaria control at the launch of Roll Back Malaria, the newly appointed RBM Director David Naborro replied: “The core of the health sector is well defined, but its boundaries are nebulous.” That was, at best, a non-committal statement of a view that would soon become reality in a health sector-confined strategy.

It is beyond any dispute that, over the past 25 years, this approach has had a substantial positive impact on malaria incidence and on under-five mortality. The lessons learned from the 1950s/60s eradication campaign, with its singular reliance on one intervention, have been taken to heart. Biological hurdles (insecticide resistance), social obstacles (community resistance against spraying) and political disinterest (re-allocation of government funds once eradication targets had been achieved) have been overcome. As a result, a multifactorial set of activities in the context of strengthening health services has born fruit. Several countries have been able to achieve a number of years without local transmission, thus qualifying for the WHO certifcation as malaria-free. Yet, over the past eight years or so there has been a discernible trend of stagnation and regression in incidence rates in several parts of the world. It was perhaps too simplistic or naïve to assume that a two-pronged health-sector confined approach would be able sustain a process of progressive realisation of malaria elimination towards full eradication in the face of a complex and well-entrenched biological system. The recent invasion of Africa by *Anopheles stephensi* once again shows how changes in natural and manmade pressures can result in important adjustments in the environmental and social determinants of malaria transmission. Since the 1990s we have effective instruments to assess hazards and risks as part of development planning for infrastructure projects (energy, agriculture, transport, urbanisation), and we also have a range of environmental engineering and management techniques at our disposal that can reduce those risks, creating a more resilient basis for health sector interventions. But in the current global strategy there has been little or no room for this. And while it is true that vaccination against malaria is now finally on the horizon, its relative role in the overall integrated disease control approach is yet to be established.

The 1980s and 1990s witnessed important efforts to promote intersectoral action in support of reducing the transmission risks of malaria and other vector-borne diseases. The objectives of an interagency expert group (the WHO/FAO/UNEP Panel of Experts on Environmental Management for Vector Control, later joined by UN-Habitat) included the promotion of health impact assessment of water resources development projects as a basis for the conceptualisation, design and implementation of malaria prevention and control measures as part of the planning, construction and management of such projects. The arrangements between the UN agencies set a clear example of the intersectoral arrangements governments could adopt to optimize intersectoral action. There were clear roles and responsibilities for all sectors. With the advent of the RBM initiative this intersectoral dimension of an IVM approach was side-lined. Now, in the 2020s, the idea of resilience has emerged convincingly. It has become the keyword in discussions over climate change, the One Health concept and planetary health. For malaria, the interpretation of the word resilience has so far been narrowed down to discussions over the resilience of malaria control programmes themselves, not over the resilience of the impact of integrated interventions in different and changing eco-epidemiological settings. Sustaining the important achievements of the past decades, ensuring that malaria-free countries stay malaria free and deploying resources so that the final remains of malaria in the world – the places where it will be the most difficult to eliminate (reference is made to the challenges at the end of the WHO smallpox campaign)- will indeed disappear - all this will require an adoption of the concept of resilience in its widest sense.

It is now time to revisit earlier concepts, to develop and strengthen new ones and to provide sustainable malaria prevention and control with a broader foundation aiming for truly resilient results. The need to promote institutional arrangements conducive to intersectoral action is as great in WHO Member States as it is within the structure of the World Health Organization itself.
